# Mechanical Property of Polypropylene Gels Associated with That of Molten Polypropylenes

**DOI:** 10.3390/gels7030099

**Published:** 2021-07-23

**Authors:** Tetsu Ouchi, Misuzu Yamazaki, Tomoki Maeda, Atsushi Hotta

**Affiliations:** Department of Mechanical Engineering, Keio University, 3-14-1, Hiyoshi, Kohoku-ku, Yokohama 223-8522, Japan; tetsu.ouchi@duke.edu (T.O.); ymisuzu332@gmail.com (M.Y.); tomoki.maeda.polymer@vc.ibaraki.ac.jp (T.M.)

**Keywords:** syndiotactic polypropylene, isotactic polypropylene, gel, melt

## Abstract

This study aims to understand the fundamental mechanical relationship between polypropylene (PP)-gels and solid PPs without solvent through mechanical and thermal analyses, by which the mechanical similarities between molten PPs and PP gels were found, leading to the reliable estimate of the mechanical properties of semi-crystalline gels. The gelation of syndiotactic and isotactic polypropylenes (sPP and iPP) was found when PPs were dissolved in 1,2,3,4-tetrahydronaphthalene (tetralin). Interestingly, it was found that the storage modulus of sPP-gel became higher than that of iPP-gel at low PP concentration (<~40 wt%). The result was distinctly different from the result of neat solid PPs (without solvent), where the modulus of solid sPP is generally significantly lower than that of solid iPP. Such inversion behavior in the mechanical property of semi-crystalline gels had not been reported and discussed before. By further investigation of the storage moduli of neat sPP and iPP, it was found that the storage modulus of sPP became higher than that of iPP above the melting points of PP, which was similar to the behavior of the storage moduli observed in the diluted PP-gels. Such similarity between PP-gels and PP melts was also observed within iPP samples with different molecular weights.

## 1. Introduction

Polymeric gels are widely used materials for practical applications such as sensors, dampers, and contact lenses [1,2,3,4,5]. Among the polymeric gels, physical gels have recently gained attention since they have the potential to be effectively used as e.g., eco-materials by remoulding and reusing the gels. Such thermal reversibility of the physical gels can be largely attributed to their relatively low intermolecular forces including van der Waals force and hydrogen bonding. Physical gels therefore demonstrate reversible sol-gel transitions, which can hardly be observed in ordinary chemical gels.

Semi-crystalline polymers can form efficient molecular crosslinks that can lead to the construction of physical gels [6,7,8,9,10,11,12,13]. The semi-crystalline gels can also provide a thermoreversible sol-gel transition, which is owed largely to the thermoreversible characteristics of the crystalline structures, where the crystalline crosslinks or the agglomerations of the conformational molecules would melt at high temperature, generally right above the melting points of the crystals. There have already been some reports on semi-crystalline gels regarding sol-gel transitions investigated by other groups. For example, it was concluded that syndiotactic polystyrene (sPS) in naphthalene formed a gel that transformed into sol by heating, which was thermoreversible (i.e., the sol transformed into gel by cooling) [6]. Syndiotactic poly(methyl methacrylate) (sPMMA) in toluene and polysaccharide in water also presented a thermoreversible sol-gel transition by controlling temperature [9,14,15].

The mechanical property of polymers is strongly related to the microstructures of the polymers [16,17,18]. The mechanical properties of semi-crystalline gels are not well understood, although some structural studies have already been reported in connection with the mechanical properties of the semi-crystalline gels. Daniel et al. and De Rudder et al. related the modulus of sPS-gel to the molecular conformations, revealing that 3D networks of sPS-gels with helical crystalline structures showed higher storage modulus (G’) than the 3D networks of sPS-gels with trans-planar crystalline structures [19]. It was also reported that G’ of sPS in bromoform increased as the molecular structures of sPS changed from random coils, through helical structures (although occasionally), to dimers of helices simultaneously constructing sPS-gel by forming 3D networks [20]. Similar results could be found in sPMMA in toluene and poly(vinylidene fluoride) in ethylene carbonate [9].

Polyolefins are the most widely used semi-crystalline polymers by which semi-crystalline gels are obtained [21,22,23]. The structural analysis of the polyolefin-gels has been extensively investigated by several groups [24,25,26,27,28,29,30]. Nakaoki et al. studied isotactic polypropylene (iPP)/*o*-dichlorobenzene gel that formed imperfect α-iPP crystalline structure with 3/1 helical molecular conformation, which eventually made crosslinks of a 3D network [31]. The group also studied the crosslinking structure of syndiotactic polypropylene (sPP)/*o*-dichlorobenzene gel with TTGG conformation that was different from the conformation observed in a solid sPP crystal [32]. As for the mechanical properties of polyolefin-gels, limited research has been carried out thus far [1,33]. Wang et al. studied sPP-b-EPR-b-sPP triblock gel dissolved in mineral oil at a weight concentration of 6% and found that at larger strains after the step-cycle processing, the tangent modulus became three times higher than that at lower strains [1].

In this study, PP-gels and solid PPs without solvent were investigated with the aim of understanding the fundamental mechanical relationship between them through mechanical and thermal analyses. It was found that the dynamic mechanical property of the neat PPs in the melt state closely resembled that of the PP-gels at low concentration. The results indicate that the amorphous region of the PP-gels brought about a decisive effect on the mechanical properties of PP-gels at low PP concentration (<~40 wt%), while the crystalline structures had a major impact on the mechanical features of PP-gels at high PP concentration (>~40 wt%). The empirical finding well explains the interesting facts that the modulus of sPP-gel was higher than the modulus of iPP-gel, whereas the modulus of neat solid sPP was lower than the modulus of neat solid iPP. The similar trend was also found in the experiments of iPP-gels and neat iPP-solid samples with different molecular weights.

## 2. Results

### 2.1. Mechanical Properties of iPP and sPP in the Solid and the Gel States

**Figure 1:**
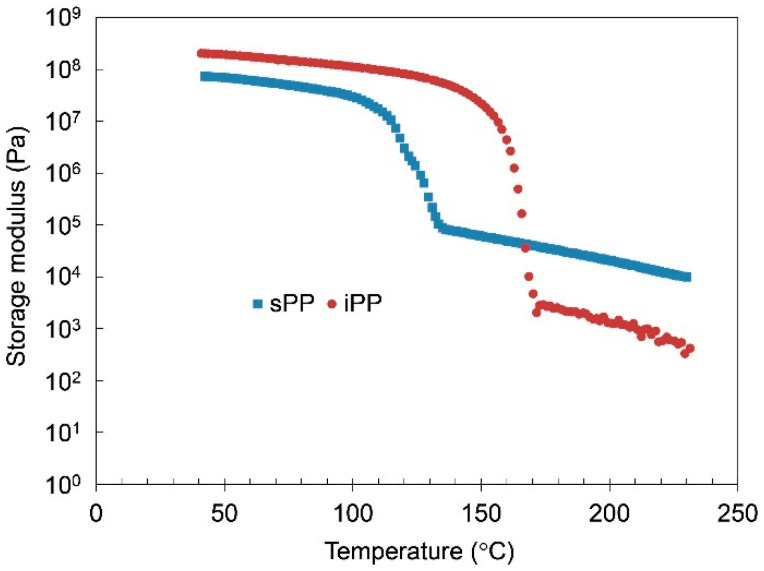
Storage modulus of polypropylene (PP)-solids as a function of temperature.

Dynamic mechanical analyses (DMA) of iPP and sPP were performed to study the difference in the mechanical properties between iPP and sPP. Figure 1 shows the results of DMA for iPP-solid with Mw = 190,000 and sPP-solid with Mw = 174,000. From the DMA results of PP-solids, it was found that, almost up to the melting point of PPs, the storage modulus G’ of iPP was overall considerably higher than that of sPP. In fact, the G’ of iPP-solid was about three times as high as that of sPP at 40 °C. The DMA data also showed the melting points (T_m_) of iPP and sPP at 167.2 °C and 145.4 °C, respectively. However, at the temperature above the melting points (T > T_m_), the storage modulus of iPP-melt became lower than that of sPP-melt. The results indicate that the storage modulus of amorphous molten sPP was higher than that of amorphous molten iPP, because above T_m_ both PP specimens became non-crystalline to be isotropic amorphous. It should be noted that iPP and sPP had almost the same molecular weights in this experiment, so the difference in the molecular weights should be negligible.

**Figure 2:**
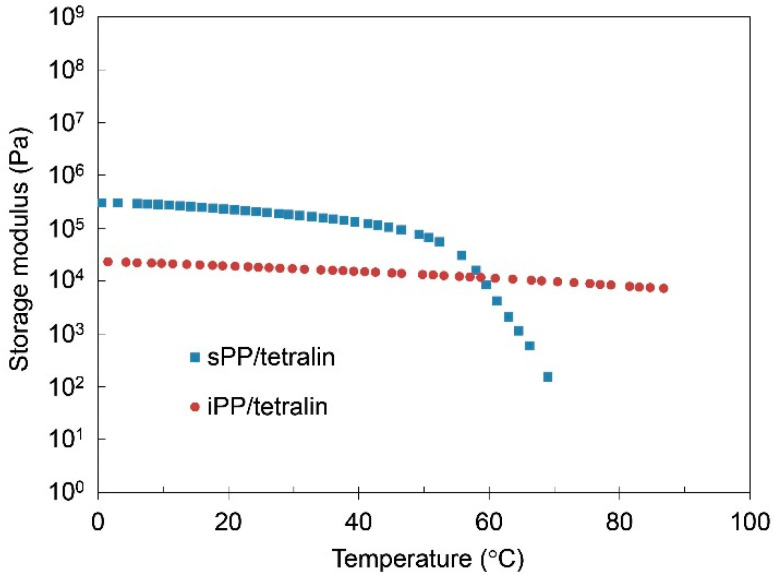
Storage modulus of PP/tetralin10 gels as a function of temperature.

Figure 2 shows the results of G’ of PP-gels (PP/tetralin10), showing that the G’ of sPP-gel was higher than that of iPP gel below the gel-sol transition temperature. The gel-sol transition temperature (T_gel_) was observed at relatively low temperature of ~70.5 °C by the DMA results (cf. ~65.2 °C by DSC) and ~110 °C by DSC results for sPP-gel and iPP-gel, respectively, which were both significantly lower than the melting temperatures of neat PPs. The root of the gel-sol transition was in fact the melting of the crystalline structures. It was also found that the storage modulus of PP-gels below T_gel_ resembled the results of PP-solid above T_m_, where G’ of sPP was higher than that of iPP (Figure 1). Furthermore, the results where G’ of sPP-gels were higher than that of iPP-gels could not be simply explained by the change in the crystalline structures (i.e., crystalline conformations) because FTIR results showed that the crystalline structures of iPP and sPP-gels were both quite similar to those of iPP and sPP solids, respectively (Figures S1 and S2 in the Supplementary Materials): iPP-gel and iPP-solid formed mainly α crystal, while sPP-gel and sPP-solid consisted of mainly Form I. It was therefore surmised that the mechanical characteristics of PP-gels was decisively controlled by the amorphous regions of the PPs.

### 2.2. Concentrations of iPP and sPP

**Figure 3:**
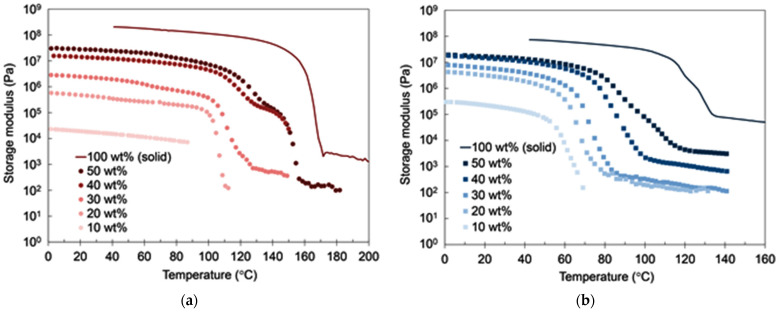
Storage modulus of PP-gels with different PP concentrations as a function of temperature: (**a**) isotactic polypropylene (iPP) (Mw = 190,000)/1,2,3,4-tetrahydronaphthalene (tetralin) gels; (**b**) syndiotactic polypropylene (sPP) (Mw = 174,000)/tetralin gels.

**Figure 4:**
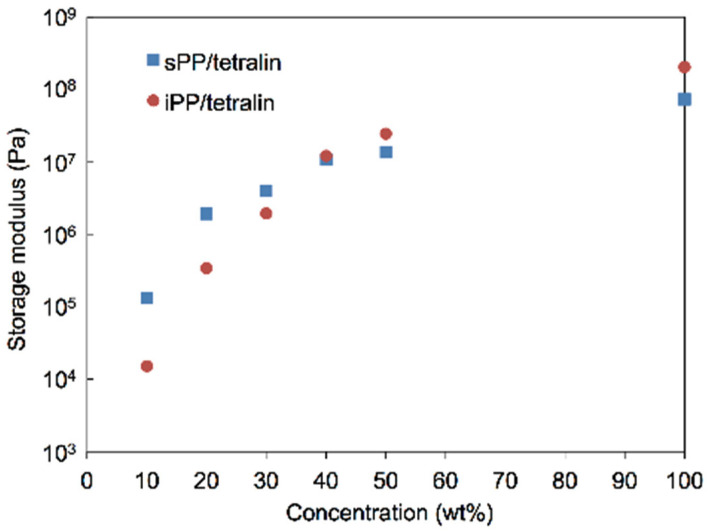
Storage modulus of PP-gels at 40 °C as a function of the concentration of PP.

**Figure 5:**
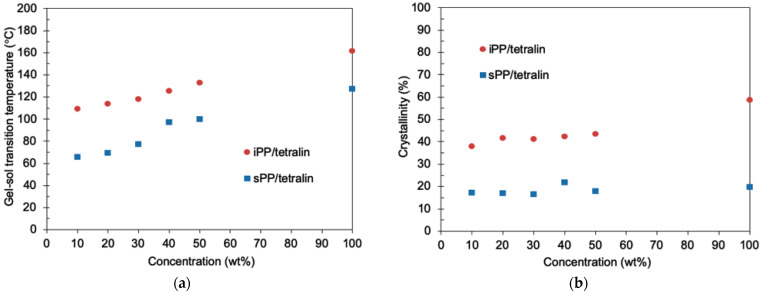
(**a**) The gel-sol transition temperature and (**b**) the crystallinity of PP as a function of PP concentration.

To study the effects of PP concentrations on the mechanical and the thermal properties of PP-gels, DMA and different scanning calorimetry (DSC) analysis were carried out by changing the concentrations of PPs. Figure 3a,b show the DMA results of iPP-gels and sPP-gels, respectively, with different PP concentrations. Each curve represents the storage modulus G’ as a function of temperature. It was found that G’ was increased by increasing the content of PPs below the gel-sol transition temperature for both iPP and sPP specimens. Accordingly, the G’ at the PP concentration of 50 wt% became closer to the G’ of PP-solid. Figure 4 shows the results of G’ of iPP and sPP samples as a function of PP concentration in 1,2,3,4-tetrahydronaphthalene (tetralin) at 40 °C. The inversion observed in the mechanical properties of iPP and sPP was detected right at the PP concentration of ~40 wt%. As mentioned above (Figure 1 and Figure 2), PP-gels at lower PP concentrations (<~40 wt%) behaved as molten PP-solid above T_m_, where G’ of sPP was higher than that of iPP, due to the dominant influence of the amorphous phases to the mechanical properties. However, as the concentration increased, the influence of the crystalline structures became more dominant. Therefore, PP-gels at higher PP concentrations (>~40 wt%) behaved as PP-solids below T_m_, where G’ of iPP was higher than that of sPP. In summary, the results indicate that the amorphous region of PPs had direct influence on the mechanical properties of PP-gels at lower PP concentrations (<~40 wt%), whereas the crystalline region of PPs had a major impact on the mechanical properties of PP-gels at higher PP concentrations (>~40 wt%). The thermal properties of PP-solids and PP-gels were conducted by DSC. Figure 5a shows the relationship between the gel-sol transition temperature T_gel_ and the PP concentrations. It was found that T_gel_ increased gradually and almost linearly as the PP concentration increased up to the melting temperatures (T_m_) of neat PPs without solvent. The experimental results indicate that the root of the gel-sol transition was in fact the melting of the crystalline structures of PPs. The shift of the gel-sol transition temperatures was also reported in polyethylene with decahydronaphthalene (decalin), toluene, *o*-xylene, benzene, and tetralin, and also in iPP with decalin, toluene, *o*-xylene, and tetralin studied by Matsuda et al. [24,34].

Figure 5b shows the relationship between the crystallinity and the concentration of PPs. Regarding crystalline conformations, FTIR results showed that iPP-gel and iPP-solid were both formed mainly by α crystal and that sPP-gel and sPP-solid were both made of Form I (Figures S1 and S2). The enthalpies of 100% α-crystal and Form I were therefore used for the calculation of the crystallinity of PP-gels and PP-solids. As Figure 5b shows, the crystallinity of iPP/tetralin gels was almost constant (~40%) or slightly increased in the gel state regardless of the increase in the iPP concentration. The crystallinity of the solid iPP was higher than that of iPP/tetralin gels, which implies that the crystallinity of iPP was affected by the surrounding solvent (tetralin) of iPP, even if the solvent concentration was relatively low. The crystallinity of sPP/tetralin gels also remained unchanged (~20%) up to even 100 wt% (sPP-solid), suggesting that the crystallinity of sPP-gels was undisturbed by the surrounding solvent. T_gel_ and the crystallinity generally relate to the intermolecular force, the crystalline perfection, and the amount of the crystalline structure. Nakaoki et al. reported that iPP-gel in *o*-dichlorobenzene, which had incomplete crystalline crosslinks, presented lower T_gel_ and ΔH_gel_ (i.e., crystallinity) [11]. It was surmised that for both iPP and sPP-gels, the intermolecular force between PP molecules may have become lower (the molecular mobility became higher) as PP absorbed more solvent, and that the crystalline structure may have become incomplete with lower crystallinity as the PP concentration decreased. All these modest experimental results of the crystalline structures by DMA and DSC indicate that the crystalline region of PP had only a gradual or subtle effect on the drastic change in the mechanical properties of PP-gels. Moreover, it is reasonably expected that the PP-gels at low PP concentrations (<~40%) had relatively small “crosslinking” domain of the crystalline structures, while, for PP-gels at high PP concentration, the crystalline structures should have become the matrix of the gel, where crystalline features of PP governed the characteristics of the PP-gels. Therefore, at lower PP concentrations, it can be surmised that amorphous regions had a significant influence on the characteristics of the PP-gels.

### 2.3. Molecular Weight of iPP

**Figure 6:**
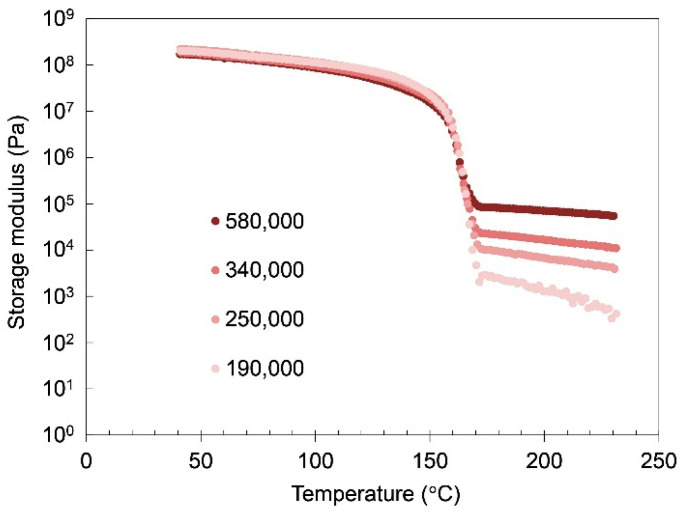
Storage modulus of iPP-solids with different molecular weights as a function of temperature.

**Figure 7:**
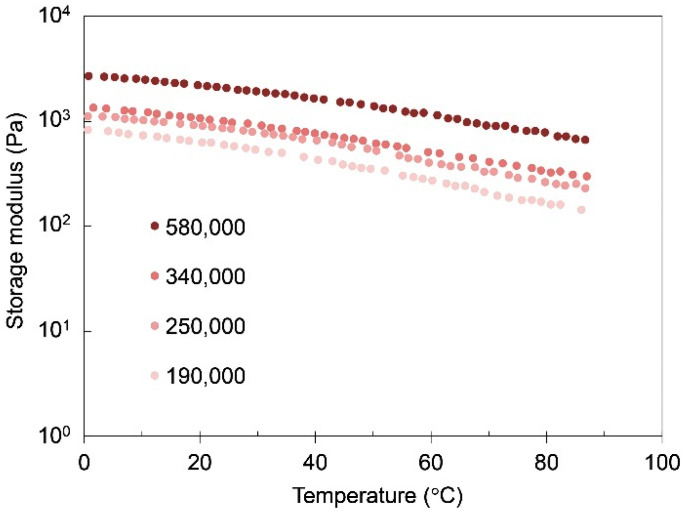
Storage modulus of iPP/tetralin5 gels with different molecular weights of iPP as a function of temperature.

**Figure 8:**
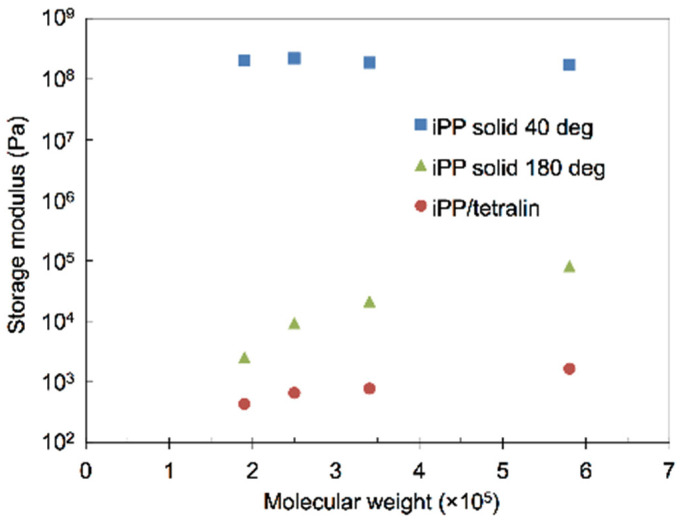
Storage modulus against molecular weights of iPP/tetralin5 at 40 °C, iPP-solids at 40 °C, and iPP-solids at 180 °C.

The effect of the molecular weight on the mechanical properties of iPP-solid and iPP-gel was studied to confirm the experimental results of the molten PPs and PP-gels discussed thus far. Since the molecular weight of polymers is closely related to the molecular entanglement of polymers, it is expected that the molecular weight has a direct effect on the mechanical properties of gels as well as solid polymers. Figure 6 shows the effect of the molecular weight on the viscoelasticity of iPP-solid. Below T_m_ of iPP (~160 °C), the molecular weight had not much effect on the storage modulus G’ of iPP, whereas above T_m_ of iPP, the storage modulus G’ of molten iPP became higher when the molecular weight of iPP was higher. The G’ above T_m_ generally reflected the molecular mobility due to the entanglement of the targeted polymers and the G’ can be roughly estimated by G’~nkT where *n* is the number of entanglement per volume. The results indicate that the molecular chains became more entangled above T_m_ as the molecular weight of the iPP became higher, which is plausible considering the nature of the polymeric molecular chains. Figure 7 shows the molecular weight dependence of the iPP/tetralin gels on the storage modulus at 5 wt%. Here, the concentration of 5 wt% was chosen to make the molecular weight dependence more prominent, as indicated in Figure 4, where the amorphous phase became more dominant with the decrease in the concentration. At all temperatures the iPP-gel in tetralin showed the strong dependence on molecular weight as observed in the temperature scan in iPP-solid above T_m_. G’ became higher in the order of molecular weights from lowest (190,000 g/mol) to highest (580,000 g/mol). The trend closely resembled that of the storage moduli of iPP with different molecular weights above T_m,_ where G’ of iPP increased with the increase in the molecular weight of iPP. It was considered from these results that the modulus of the iPP-gel was approximately logarithmically proportional to the amount of the molecular entanglement in the amorphous structures of the iPP, while the crosslinks of the gel were due to the crystalline structures of the iPP. Figure 8 shows the relationship between G’ and the molecular weights of iPP-gels and iPP-solid. For the iPP-gels, G’ at 40 °C was selected and plotted in Figure 8. Both G’ of iPP-gels and molten iPP (iPP-solid at 180 °C) presented similar characteristics in the graph, showing higher G’ at higher molecular weight. Since crystalline structures should not exist in the molten iPP, the results demonstrated that the mechanical properties of the dilute iPP were highly dependent on the mechanical properties of the amorphous structure of iPP.

### 2.4. PP-Gels with Other Solvents

**Figure 9:**
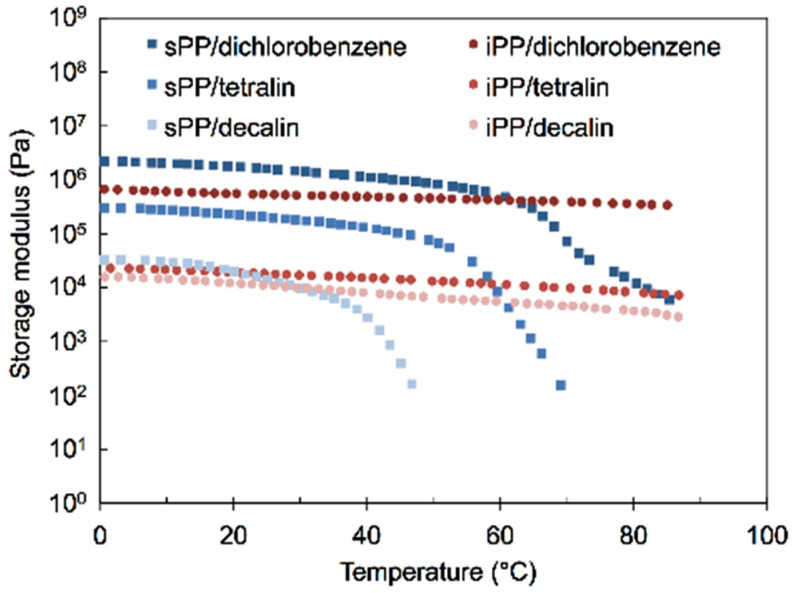
Storage moduli of PP/tetralin10, PP/decalin10, and PP/dichlorobenzene10 gels as a function of temperature.

The same tendency was confirmed by PP-gels with other solvents such as decahydronaphthalene (decalin) and *o*-dichlorobenzene. Figure 9 shows the DMA results of PP-gels at a concentration of 10 wt% with tetralin, decalin, and *o*-dichlorobenzene. The results showed that the G’ of sPP-gels was higher than that of iPP-gels, exhibiting the same trend as observed in the relationship between PP/tetralin gels at low concentration and PP-solid above T_m_. The results also supported the idea that at low concentration, the mechanical properties of PP-gels are conclusively determined by the amorphous regions of PP, indicating that the experimental results could be universal and independent of the types of solvents.

## 3. Discussion

There have been several reports that have connected semi-crystalline gel features with the amorphous region of the target semi-crystalline polymer. It is also generally recognised that semi-crystalline polymers with solvent are more flexible than the original solid polymers [35,36,37,38,39]. Gowd et al. reported that sPS solvent complex with a higher content of solvent showed a lower transition temperature for the crystalline structure [38]. They considered that solvent molecules in the amorphous regions were important for the enhancement of the molecular mobility, eventually reducing the transition temperature of the crystalline structure. Yoshioka et al. also studied the glass transition temperature of sPS from the amorphous region of sPS, and reported that sPS with chloroform, benzene, or toluene exhibited a drastic decrease in the glass transition temperature [36]. The reports concluded that polymers with solvents, including gels, had lower density of 3D networks with higher mobility of amorphous molecular chains than the solid polymers without solvents due to the plasticising effect of the solvents. From the DSC results in this study, it was also found that T_gel_ increased and the crystallinity slightly increased (while the crystallinity of sPP-gels remained almost constant) as the concentration of PP increased. The results implied that at low concentration (<~40 wt%), the crystalline structure, i.e., the crosslinks of the PP-gels, was rather small in its amount with possibly incomplete structures, leading to the construction of flexible 3D networks as was reported previously [11]. This therefore indicates that the mechanical properties of a low-concentration gel were largely dominated by amorphous regions, presenting similarity in the storage modulus to the amorphous molten phase in solid PP. The results were also supported by the report by Nakaoki et al. that sPP/*o*-dichlorobenzene gels showed relaxation times typical of the rubbery phase in sPP, instead of the ordinary crystalline phase with TTGG molecular conformations [32].

At a high concentration (>~40 wt%), in contrast, as mentioned above, G’ of PP-gels was similar to that of solid PP below T_m_, where G’ of iPP was higher than that of sPP. The results can be strongly attributed to the increasing influence of the crystalline structures in the gel due to the increase in PP concentration. Thus, the characteristics of PP-gels with higher concentrations of PP became closer to the solid PP below T_m_ with dominating crystalline regions. Actually, both in solid and in gel at a higher PP concentration, a larger amount of crystalline region restricted the mobility of amorphous molecular chains by losing its rubber elasticity. Hence, the mechanical properties of PP-gels at higher PP concentration were mainly dominated by the mechanical properties of solid crystalline PP.

The similarity between polymer solution and molten polymer was first discussed long ago by Flory and Huggins [40]. They introduced the Flory–Huggins interaction parameter χ to describe the thermodynamics of polymer solution. It was generally recognised that the molecules in the polymer solution with *χ* = 0.5 (*θ* solvent) act as if they were ideal chains without solvent, simultaneously cancelling the excluded volume effects of the polymer. Such polymer solution could behave identically to the polymer in the molten amorphous phase. The results of the experiments in this study clearly indicate that the mechanical properties of dilute PP-gels could also be predicted by the mechanical properties of neat solid PP in the molten state, revealing that the above similarity could be applied to the semi-crystalline polymeric gels. In fact, *χ* can be simply defined as *χ* ~ $V{\left(\Delta \delta \right)}^{2}/T$, where Δ*δ* are the difference in the solubility parameters between solute and solvent, and *V* is the molar volume of the solvent, which should also be related to the molar volume of the polymer [41]. *χ* is therefore strongly related to the solubility parameters of the solute and the solvent. In this study, the solubility parameters of PP, tetralin, decalin, and *o*-dichlorobenzene are 8.0, 9.5, 8.8, and 10.0, respectively. *θ* solvent with *χ* = 0.5 is exactly in between good solvent and poor solvent. Therefore, the three solvents should be in between good solvent and theta solvent for PP since they readily dissolved PP. However, since *χ* is also directly related to *V* and *T*, the experimental conditions in this study may have been adjusted to the near θ solvent condition. Additionally, according to the results of the storage moduli against frequency regarding solid PP above T_m_, sPP did not behave as complete viscous melt (where G’ and G” were not well fitted to ~ω^2^ and ~ω, respectively, which most ideal polymeric viscous melt should follow), whereas iPP almost behaved as viscous flow. The results simply indicate that there existed non-negligible interaction between sPP molecules even above T_m_ of PP, which resulted in the enhancement of the storage modulus of sPP. Nevertheless, the experimental results could be applicable to the estimation of the mechanical properties of semi-crystalline gels by investigating the amorphous phase of the solid semi-crystalline polymers used for the gels at lower polymer concentrations. Further studies would be needed to confirm the generality of this estimation.

## 4. Conclusions

The mechanical analyses of solid, melted, and gel states of PPs were carried out. From the DMA results, it was confirmed that, below T_m_, the G’ of solid iPP was higher than that of solid sPP, while on the other hand, above T_m_, the G’ of solid iPP was lower than that of solid sPP. The results indicate that the G’ of amorphous regions of sPP was higher than that of iPP. Regarding the DMA results of the PP-gels at the low concentration of PP (<~40 wt%), the G’ of iPP-gel was found to be lower than that of sPP-gel. At the high concentration of PP (>~40 wt%), the G’ of iPP-gel became higher than that of sPP-gel. The mechanical results of dilute PP-gels intriguingly resembled the results of solid PPs above T_m_, when PPs were in the amorphous state. The relatively concentrated PP-gels resembled solid PPs below T_m_, when PPs were in the semi-crystalline phase. Such mechanical similarity between PP-gels and molten PP was also observed in the iPP samples with different molecular weights. From the structural point of view, the results indicate that the molecular mobility of the gels could stem from the amorphous regions of the semi-crystalline PP, while the stable crosslinks of the gels could be derived from the crystalline structures of the semi-crystalline PP, especially at lower concentration. The DSC analyses also confirmed the increasing crystalline features of the gels especially at higher PP concentration, presenting growing T_gel_ and the increasing crystallinity of PP-gels at a higher concentration of PPs. From these experimental results, it was concluded that the amorphous phase of PP had a profound influence on the mechanical properties of PP-gels at low PP concentration, while the crystalline phase of PP had a major impact on the mechanical properties of PP-gels at relatively higher PP concentration. The experimental findings could be widely applicable to the estimation of the mechanical properties of semi-crystalline gels in general by investigating the amorphous and the semi-crystalline phases of the solid semi-crystalline polymers constituting the gels.

## 5. Materials and Methods

### 5.1. Materials and Sample Preparation

Pure isotactic polypropylene (iPP-solid) and syndiotactic polypropylene (sPP-solid) without solvents were obtained from Aldrich, Tokyo, Japan (iPP: Mw = 190,000, 250,000, 340,000, and 580,000, sPP: Mw = 174,000). Decahydronaphthalene (decalin, Wako Pure Chemical Industries, Ltd., Osaka, Japan), 1,2,3,4-tetrahydronaphtalene (tetralin, Wako Pure Chemical Industries, Ltd., Osaka, Japan), and *o*-dichlorobenzene (Junsei Chemical Co., Ltd., Tokyo, Japan) were selected as the solvents since the boiling temperatures of the solvents (decalin: T_b_ = ~190 °C, tetralin: T_b_ = ~207 °C, and *o*-dichlorobenzene: T_b_ = ~180 °C) were all higher than the melting temperatures of the polypropylenes (iPP: T_m_ = 161.17 °C, sPP: T_m_ = 127.17 °C).

PP was added slowly into the solvents to make PP solutions before gelation. The concentration of PP was precisely controlled by the weights of PP and the solvents. The prepared PP solutions were stirred overnight at temperature ranging from 130 °C to 170 °C depending on the solubility of PP in a tightly capped vial to become homogeneous sol. The sol was then cooled to room temperature to form gel (PP-gel). The concentration of the gel was changed from 5 wt% (labelled as “iPP/tetralin5”) to 50 wt% (as “iPP/tetralin50”) for thermal and mechanical testing. All specimens were heated up to well above the melting point (T_m_) or the gel-sol transition temperature (T_gel_) of the samples to erase any thermal history, before cooling down to 0 °C (as for PP-solid, the specimen was cooled down to 40 °C) at the cooling rate of 10 °C/min to prevent the evaporation of solvents. The PP-gels were kept at 0 °C for 10 min until the gels reached their equilibrium (120 min for sPP/decalin10), which was carefully determined by DSC.

### 5.2. Mechanical, Thermal, and Structural Analysis

#### 5.2.1. Dynamic Viscoelastic Measurement

Rheological measurements of neat PP without solvents (PP-solid, i.e., 100 wt% of PP) and PP-gels were carried out using rheometer (ARES-G2, T.A. Instruments, Tokyo, Japan) to measure the storage moduli G’ of PP-solids and PP-gels. The thermal condition of the rheological measurements was basically the same as the condition mentioned above: the specimens were heated above T_m_ or T_gel_ to erase the thermal history of the specimens, and then cooled down to 0 °C (40 °C for PP-solid) at the cooling rate of 10 °C/min. After 10 min at 0 °C (120 min for sPP/decalin), the measurements started from 0 °C at the heating rate of 10 °C/min, during which the T_gel_ was observed as a crossover point of G’ and G” (i.e., tanδ = 1). As for the dilute samples (≤10 wt%), a Peltier-plate geometry was used for the rheological measurement. The maximum temperature of the rheological measurement, which can be reached by the Peltier plate in a reliable experimental setting in this study, was 85 °C. For the higher concentration samples (≥20 wt%), a metal-plate geometry with an oven was used instead of Peltier, since PP-gels with higher PP concentration had higher T_m_ or T_gel_ that could reach above 85 °C. The experiments were conducted using a stainless parallel-plate whose diameter was 25 mm. The moduli of PP-solid, however, could be significantly high, and the rheological measurement was performed using the aluminium parallel-plate geometry with a diameter of 8 mm. The strain was set at 0.01–1.5%, which was well within the linear regime of the specimen. The frequency was fixed at 1 Hz. The data below ~100 Pa were ignored due to the uncertainty of the DMA experiments.

#### 5.2.2. DSC Measurements

The thermal analyses of PP-solids and PP-gels were conducted to measure T_m_, T_gel_, and the enthalpy (ΔH) of each specimen by differential scanning calorimetry (DSC822, Mettler Toledo, Tokyo, Japan) under a nitrogen atmosphere. The sample weight was above ~25 mg, which was sufficient to measure T_m_, T_gel_, and the enthalpy (ΔH). Gel samples were contained in a medium pressure pan (medium pressure/Viton 120 μL) and PP-solid was contained in an aluminium pan (aluminium standard 40 μL). Specimens were all heated at 10 °C/min from 25 °C to the temperature T_high_ that was well above T_m_ or T_gel_ of each specimen to erase the thermal history of the specimens. Then, the samples were cooled down to 0 °C (40 °C for PP-solid) at the temperature rate of 10 °C/min and held at 0 °C for 10 min (120 min for sPP/decalin). Then the DSC measurement started from 0 °C to T_high_ at a heating rate of 10 °C/min, during which the gel-sol transition temperature T_gel_ was observed as an endothermic peak temperature. The measured enthalpy (ΔH) was converted to the gel-sol enthalpy ΔH_gel_ by dividing ΔH by the total weight of the gel that was multiplied by the weight fraction of the solid PP. Thus, ΔH_gel_ can be defined as “ΔH per PP gram”, with which the enthalpy of gels and solids can be compared. The crystallinity can also be calculated by further dividing ΔH_gel_ by ΔH_max_. ΔH_max_ is the melting enthalpy of PP with 100% crystallinity. Amounts of 178 J/g (α crystal) and 183 J/g (Form I) were used for the ΔH_max_ of iPP and sPP, respectively [42,43,44].

#### 5.2.3. FTIR Analysis

FTIR analysis was carried out using FTIR (FTIR-4200, JASCO, Tokyo, Japan) with CaF_2_ glass to identify the crystalline and the conformation structures of the PP specimens. The wavenumber resolution was 2.0 cm^−1^. Since the direct FTIR spectra of PP-gels contain the signal of solvent, spectral subtraction analysis was conducted to identify the crystalline and the conformational structures of PPs in gel. Differential spectra were established by eliminating solvent spectra by adjusting 1037 cm^−1^ peak to become 0 (baseline). Spectra were then analysed from the wavenumber of 1350 cm^−1^ to 900 cm^−1^.

## Data Availability

The data generated or analyzed during this study are available from the corresponding author on reasonable request.

## Supplementary Materials

The following are available online at https://www.mdpi.com/article/10.3390/gels7030099/s1, Figure S1: FTIR spectra of iPP/tetralin 10, tetralin, differential spectrum of iPP/tetralin 10—tetralin, and iPP. Figure S2: FTIR spectra of sPP/tetralin 10, tetralin, differential spectrum of sPP/tetralin 10—tetralin, and sPP.

## References

[1] Wang Z., Niu Y., Fredrickson G.H., Kramer E.J., Shin Y.-W., Shimizu F., Zuo F., Rong L., Hsiao B.S., Coates G.W. (2010). Step-Cycle Mechanical Processing of Gels of sPP-b-EPR-b-sPP Triblock Copolymer in Mineral Oil. Macromolecules.

[2] Haraguchi K., Takehisa T., Fan S. (2002). Effects of Clay Content on the Properties of Nanocomposite Hydrogels Composed of Poly(N-isopropylacrylamide) and Clay. Macromolecules.

[3] Chirila T.V., Hong Y., Dalton P.D., Constable I.J., Refojo M.F. (1998). The use of hydrophilic polymers as artificial vitreous. Prog. Polym. Sci..

[4] Kavanagh G.M., Ross-Murphy S.B. (1998). Rheological characterisation of polymer gels. Prog. Polym. Sci..

[5] Okayama Y., Nakahara K., Arouette X., Ninomiya T., Matsumoto Y., Orimo Y., Hotta A., Omiya M., Miki N. (2010). Characterization of a bonding-in-liquid technique for liquid encapsulation into MEMS devices. J. Micromech. Microeng..

[6] Malik S., Rochas C., Démé B., Guenet J.M. (2005). Thermoreversible Gelation of Syndiotactic Polystyrene in Naphthalene. Macromol. Symp..

[7] Daniel C., Deluca M.D., Guenet J.M., Brulet A., Menelle A. (1996). Thermoreversible gelation of syndiotactic polystyrene in benzene. Polymer.

[8] Malik S., Rochas C., Guenet J.M. (2006). Thermodynamic and Structural Investigations on the Different Forms of Syndiotactic Polystyrene Intercalates. Macromolecules.

[9] Buyse K., Berghmans H., Bosco M., Paoletti S. (1998). Mechanistic Aspects of the Thermoreversible Gelation of Syndiotactic Poly(methyl methacrylate) in Toluene. Macromolecules.

[10] Buyse K., Berghmans H. (2000). Thermoreversible gelation of solutions of isotactic poly(methyl methacrylate) in 2-butanone. Polymer.

[11] Nakaoki T., Inaji Y. (2002). Molecular structure of isotactic polypropylene formed from homogeneous solution. Gelation and crystallization. Polym. J..

[12] Miyazaki M., Maeda T., Hirashima K., Kurokawa N., Nagahama K., Hotta A. (2017). PEG-based nanocomposite hydrogel: Thermoresponsive sol-gel transition controlled by PLGA-PEG-PLGA molecular weight and solute concentration. Polymer.

[13] Nagahama K., Oyama N., Ono K., Hotta A., Kawauchi K., Nishikata T. (2018). Nanocomposite injectable gels capable of self-replenishing regenerative extracellular microenvironments for in vivo tissue engineering. Biomater. Sci..

[14] Morris E.R., Nishinari K., Rinaudo M. (2012). Gelation of gellan—A review. Food Hydrocoll..

[15] Kim I.Y., Iwatsuki R., Kikuta K., Morita Y., Miyazaki T., Ohtsuki C. (2011). Thermoreversible behavior of ĸ-carrageenan and its apatite-forming ability in simulated body fluid. Mater. Sci. Eng. C.

[16] Clarke S.M., Hotta A., Tajbakhsh A.R., Terentjev E.M. (2002). Effect of cross-linker geometry on dynamic mechanical properties of nematic elastomers. Phys. Rev. E.

[17] Hotta A., Terentjev E.M. (2001). Long-time stress relaxation in polyacrylate nematic liquid crystalline elastomers. J. Phys. Condens. Matter.

[18] Hotta A., Terentjev E.M. (2003). Dynamic soft elasticity in monodomain nematic elastomers. Eur. Phys. J. E.

[19] Daniel C., Alfano D., Guerra G., Musto P. (2003). Physical Gelation of Syndiotactic Polystyrene in the Presence of Large Molar Volume Solvents Induced by Volatile Guests of Clathrate Phases. Macromolecules.

[20] De Rudder J., Berghmans H., De Schryver F.C., Bosco M., Paoletti S. (2002). Gelation Mechanism of Syndiotactic Polystyrene in Bromoform. Macromolecules.

[21] Diamanti S.J., Khanna V., Hotta A., Coffin R.C., Yamakawa D., Kramer E.J., Fredrickson G.H., Bazan G.C. (2006). Tapered block copolymers containing ethylene and a functionalized comonomer. Macromolecules.

[22] Coffin R.C., Diamanti S.J., Hotta A., Khanna V., Kramer E.J., Fredrickson G.H., Bazan G.C. (2007). Pseudo-tetrablock copolymers with ethylene and a functionalized comonomer. Chem. Commun..

[23] Deplace F., Wang Z.G., Lynd N.A., Hotta A., Rose J.M., Hustad P.D., Tian J., Ohtaki H., Coates G.W., Shimizu F. (2010). Processing-Structure-Mechanical Property Relationships of Semicrystalline Polyolefin-Based Block Copolymers. J. Polym. Sci. Part B Polym. Phys..

[24] Matsuda H., Inoue T., Okabe M., Ukaji T. (1987). Study of polyolefin gel in organic-solvents.1. structure of isotactic polypropylene gel in organic-solvents. Polym. J..

[25] Ogita T., Kawahara Y., Sawatari C., Ozaki F., Matsuo M. (1991). Morphological properties of ultrahigh molecular-weight polyethylene and low-molecular-weight polypropylene blend gel films. Polym. J..

[26] Ohta T., Ikeda Y., Kishimoto M., Sakamoto Y., Kawamura H., Asaeda E. (1998). The ultra-drawing behaviour of ultra-high-molecular-weight polypropylene in the gel-like spherulite press method: Influence of solution concentration. Polymer.

[27] Pogodina N.V., Lavrenko V.P., Srinivas S., Winter H.H. (2001). Rheology and structure of isotactic polypropylene near the gel point: Quiescent and shear-induced crystallization. Polymer.

[28] Matsuo M., Hashida T., Tashiro K., Agari Y. (2002). Phase Separation of Ultrahigh Molecular Weight Isotactic Polypropylene Solutions in the Gelation Process Estimated in Relation to the Morphology and Mechanical Properties of the Resultant Dry Gel Films. Macromolecules.

[29] Kristiansen M., Tervoort T., Smith P. (2003). Synergistic gelation of solutions of isotactic polypropylene and bis-(3,4-dimethyl benzylidene) sorbitol and its use in gel-processing. Polymer.

[30] Nakaoki T., Harada S. (2005). Melting behavior of bound solvent in isotactic polypropylene/o-dichlorobenzene gel. Polym. J..

[31] Nakaoki T., Shuto H., Hayashi H., Kitamaru R. (1998). High-resolution solid-state ^13^C n.m.r. study of isotactic polypropylene gel. Polymer.

[32] Nakaoki T., Hayashi H., Kitamaru R. (1996). Structural study of syndiotactic polypropylene gel by solid-state high resolution 13C n.m.r. Polymer.

[33] Deplace F., Scholz A.K., Fredrickson G.H., Kramer E.J., Shin Y.-W., Shimizu F., Zuo F., Rong L., Hsiao B.S., Coates G.W. (2012). Tough and Elastic Thermoplastic Organogels and Elastomers Made of Semicrystalline Polyolefin-Based Block Copolymers. Macromolecules.

[34] Matsuda H., Imaizumi M., Fujimatsu H., Kuroiwa S., Okabe M. (1984). Sol-gel transition of branched low-density polyethylene in organic-solvents. Polym. J..

[35] Rizzo P., Albunia A.R., Guerra G. (2005). Polymorphism of syndiotactic polystyrene: [gamma] phase crystallization induced by bulky non-guest solvents. Polymer.

[36] Yoshioka A., Tashiro K. (2003). Solvent Effect on the Glass Transition Temperature of Syndiotactic Polystyrene Viewed from Time-Resolved Measurements of Infrared Spectra at the Various Temperatures and Its Simulation by Molecular Dynamics Calculation. Macromolecules.

[37] Gowd E.B., Tashiro K., Ramesh C. (2009). Structural phase transitions of syndiotactic polystyrene. Prog. Polym. Sci..

[38] Gowd E.B., Tashiro K., Ramesh C. (2008). Role of Solvent Molecules as a Trigger for the Crystal Phase Transition of Syndiotactic Polystyrene/Solvent Complex. Macromolecules.

[39] Gowd E.B., Tashiro K. (2007). Effect of Solvent Molecules on Phase Transition Phenomena of Syndiotactic Polystyrene. Macromolecules.

[40] Flory P.J. (1942). Thermodynamics of High Polymer Solutions. J. Chem. Phys..

[41] Bristow G.M., Watson W.F. (1958). Cohesive energy densities of polymers. Part 1.-Cohesive energy densities of rubbers by swelling measurements. Trans. Faraday Soc..

[42] Schmidtke J., Strobl G., Thurn-Albrecht T. (1997). A Four-State Scheme for Treating Polymer Crystallization and Melting Suggested by Calorimetric and Small Angle X-ray Scattering Experiments on Syndiotactic Polypropylene. Macromolecules.

[43] Zhang X., Li R., Kong L., Wang D. (2008). Stress-induced structure transition of syndiotactic polypropylene via melt spinning. Polymer.

[44] Liu F., Guo C., Wu X., Qian X., Liu H., Zhang J. (2010). Morphological comparison of isotactic polypropylene parts prepared by micro-injection molding and conventional injection molding. Polym. Adv. Technol..

