# Supplementary material for: Mechanical Property of Polypropylene Gels Associated with That of Molten Polypropylenes
Source: Gels. 2021 Jul 23;7(3):99. doi: 10.3390/gels7030099 (PMC8395817; doi:10.3390/gels7030099)
Supplement: Supplementary file 1 [file gels-07-00099-s001.zip › gels-1253415-supplementary.pdf]

Supplementary Materials

Mechanical Property of Polypropylene Gels Associated with That of Molten Polypropylenes

Tetsu Ouchi, Misuzu Yamazaki, Tomoki Maeda and Atsushi Hotta *

Department of Mechanical Engineering, Keio University, 3-14-1, Hiyoshi, Kohoku-ku, Yokohama 223-8522, Japan; tetsu.ouchi@duke.edu (T.O.); ymisuzu332@gmail.com (M.Y.); tomoki.maeda.polymer@vc.ibaraki.ac.jp (T.M.)

* Correspondence: hotta@mech.keio.ac.jp

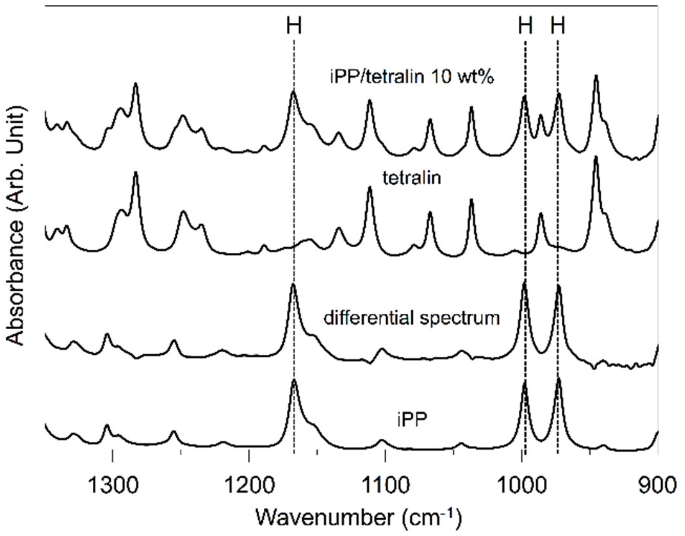

Figure S1. FTIR spectra of iPP/tetralin 10, tetralin, and iPP with the differential spectrum of iPP/tetralin10—tetralin. The FTIR spectrum of the differential spectrum and that of iPP were very similar, indicating the same configurations and structures in both specimens. H (973 cm^{-1}), H (998 cm^{-1}), and H (1167 cm^{-1}) represent helical (amorphous), helical (α crystal), and helical (α crystal), respectively.

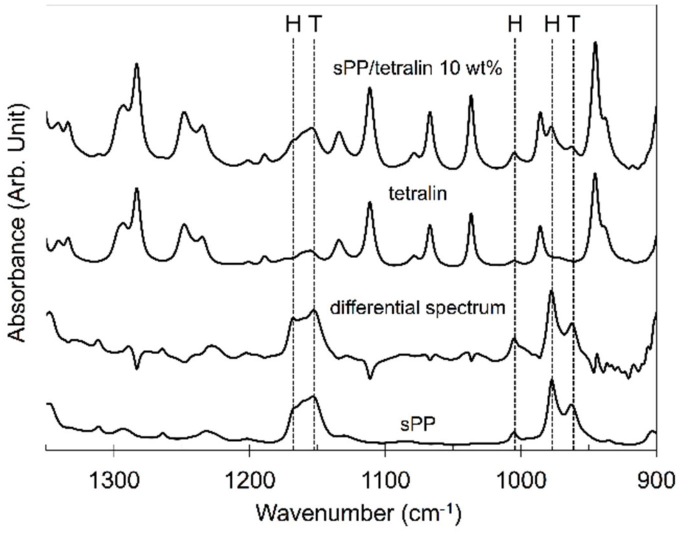

Figure S2. FTIR spectra of sPP/tetralin 10, tetralin, and sPP with the differential spectrum of sPP/tetralin10—tetralin. The FTIR spectrum of the differential spectrum and that of sPP looked very similar, indicating the same configurations and structures in the specimens. T (963 cm^{-1}), H (977 cm^{-1}), H (1005 cm^{-1}), T (1153 cm^{-1}), and H (1169 cm^{-1}) represent planar zigzag (interfacial), helical (interfacial), helical (Form I), planar zigzag (amorphous), and helical (amorphous), respectively.
